# DNAM-1/CD226 is functionally expressed on acute myeloid leukemia (AML) cells and is associated with favorable prognosis

**DOI:** 10.1038/s41598-021-97400-6

**Published:** 2021-09-09

**Authors:** Anna Chashchina, Melanie Märklin, Clemens Hinterleitner, Helmut R. Salih, Jonas S. Heitmann, Boris Klimovich

**Affiliations:** 1 Clinical Collaboration Unit Translational Immunology, German Cancer Consortium (DKTK) Department of Internal Medicine, University Hospital Tübingen, Otfried-Müller-Str. 10, 72076 Tübingen, Germany; 2 DFG Cluster of Excellence 2180 “Image-Guided and Functional Instructed Tumor Therapy (iFIT)”, 72076 Tübingen, Germany; 3 Department of Medical Oncology and Pulmonology, University Hospital Tübingen, 72076 Tübingen, Germany

**Keywords:** Cancer, Haematological cancer, Prognostic markers

## Abstract

DNAM-1 is reportedly expressed on cytotoxic T and NK cells and, upon interaction with its ligands CD112 and CD155, plays an important role in tumor immunosurveillance. It has also been reported to be functionally expressed by myeloid cells, but expression and function on malignant cells of the myeloid lineage have not been studied so far. Here we analyzed expression of DNAM-1 in leukemic cells of acute myeloid leukemia (AML) patients. We found substantial levels of DNAM-1 to be expressed on leukemic blasts in 48 of 62 (> 75%) patients. Interaction of DNAM-1 with its ligands CD112 and CD155 induced release of the immunomodulatory cytokines IL-6, IL-8 IL-10 and TNF-α by AML cells and DNAM-1 expression correlated with a more differentiated phenotype. Multivariate analysis did not show any association of DNAM-1 positivity with established risk factors, but expression was significantly associated with clinical disease course: patients with high DNAM-1 surface levels had significantly longer progression-free and overall survival compared to DNAM-1^low^ patients, independently whether patients had undergone allogenic stem cell transplantation or not. Together, our findings unravel a functional role of DNAM-1 in AML pathophysiology and identify DNAM-1 as a potential novel prognostic maker in AML.

## Introduction

Acute myeloid leukemia (AML) is the most common form of acute leukemia in adults^1^. Diagnostic classification of AML is based on morphologic, immunophenotypic, cytogenetic and molecular genetic analyses^2,3^. Rapid and accurate risk stratification is essential for guiding treatment decisions and predicting therapy outcomes, and novel prognostic markers may thus improve clinical management of patients. So far, immunophenotyping of AML blasts plays a great role in diagnosis but is less important for risk assessment. Besides typical myeloid surface markers, abnormal expression of various markers of non-myeloid lineages on AML blasts has been reported^4,5^.

DNAX accessory molecule-1 (DNAM-1, CD226) is a transmembrane nectin-like glycoprotein of the immunoglobulin superfamily and acts as an activating receptor on NK cells and T cells. Its ligands, CD112 and CD155, are likewise members of the nectin family, and the DNAM-1-CD112/CD155 axis is mainly known for its important role in NK cell-mediated killing of tumor cells^6^. Besides the well documented role of DNAM-1 in lymphocytes, expression was also reported for cells of the myeloid lineage like monocytes and platelets, where it mediates adhesion and migration^7,8^ However, nothing is known regarding the expression and function of DNAM-1 on malignant cells of the myeloid lineage. Notably, aberrant expression of immune receptors normally found on NK cells was previously reported for AML cells, with the Fcɣ receptors CD32, CD64 and CD16 being prominent examples^9–12^.

In this study we report that DNAM-1 is frequently expressed on AML patient cells, demonstrate that DNAM-1 stimulates the production of immunomodulatory cytokines by the leukemic cells and identified a significant correlation of DNAM-1 with disease outcome.

## Materials and methods

### Patient samples

Peripheral blood mononuclear cells (PBMC) of AML patients were isolated by density gradient centrifugation and frozen in liquid nitrogen. Informed consent was obtained from all patients in accordance with the Helsinki protocol. In this study all experimental protocols were conducted according to the guidelines and were approved by the local ethics committee of the University of Tübingen (approval number 13/2007V).

### Reagents and antibodies

DNAM-1 mAb clone 102511 and mouse IgG1 isotype control were from R&D Systems and BD Biosciences, respectively. All fluorescent antibody conjugates (CD33-BV421 clone WM53; CD34-APC clone 581; CD117-PE-Cy7 clone 104D2) were from BD Biosciences and used in 1:200 dilution, secondary goat-anti–mouse-PE was from Dako. Recombinant human CD112 or CD155 Fc-domain fusion proteins (rhCD112/rhCD155) were from BioLegend. Recombinant proteins were tested to be free of endotoxins by ENDONEXT EndoZyme II assay (bioMérieux).

### Cell culture

The following cell lines were obtained from German Collection of Microorganisms and Cell Cultures (Deutsche Sammlung von Mikroorganismen und Zellkulturen, DSMZ): KG-1, EOL-1, Kasumi-1, K562, HL-60, TF-1 and U937. Authenticity was routinely determined by validating the immunophenotype described by the provider using flow cytometry. Cells were cultured in RPMI1640 medium (Gibco) supplemented with 10% of heat-inactivated fetal bovine serum (PANBiotech) and 1% penicillin–streptomycin (Lonza).

### Flow cytometry

DNAM-1 surface expression was assessed by staining with a specific antibody or isotype control followed by incubation with goat-anti–mouse IgG-PE. Samples were analyzed using FACSCanto II instrument (BD Biosciences).

Leukemic cells in patient samples were first selected by FSC/SSC and then gates were applied according to the surface markers based on the individual immunophenotype defined upon routine diagnosis (CD33, CD34 or CD117). Dead cells were excluded based on 7-aminoactinomycin staining (7-AAD, BD Biosciences). Specific fluorescence indices (SFI) were calculated by dividing median fluorescence values measured with specific antibody by median fluorescence values obtained with isotype control.

### Quantitative PCR

Total RNA was isolated from 1 × 10^6^ AML cells using High Pure RNA kit (Roche). cDNA was synthesized from 500 to 1000 ng RNA using Go Script Reverse Transcription System (Promega) according to the manufacturer's instructions. DNAM-1 cDNA was amplified using 2 × qPCR SyGreen Mix (PCR Biosystems) using primers 5′-GTGGAGTGGTTCAAGATCGGG-3′ and 5′-GCTTCCTTATGACCATGCCAT-3′ (79 bp). Reference gene (GAPDH) was amplified using primers 5′-GAGTCAACGGATTTGGTCGT-3′ and 5′-TTGATTTTGGAGGGATCTCG-3′ (225 bp). PCR was performed using LightCylcer-480 instrument (Roche). Amplicons were additionally analyzed by gel electrophoresis.

### Measurements of cellular viability and cytokine production

For stimulation with DNAM-1 ligands, 96-well cell culture plates (Greiner Bio-One) were coated with 10 µg/ml solution of rhCD112, rhCD155 or hIgG1 in PBS overnight. Afterwards wells were washed with PBS and freshly thawed AML cells were plated in concentration 2 × 10^6^ cells/ml. Cytokines in supernatants from cultured AML blasts were quantified by ELISA according to the manufacturer's instructions using OptEIA assays from BD Pharmingen. Viability was measured using CellTiter-Glo® Luminescent Cell Viability Assay (Promega) according to the manufacturer's instructions.

### Statistical analysis

Statistical analysis was conducted using GraphPad Prism 8.1.0 and JMP® Pro (SAS Institute Inc., Version 14.2) software. The two-tailed unpaired Mann–Whitney or Kruskal–Wallis tests were used to compare individual groups. For comparison of DNAM-1 expression in CD34^+^ versus CD34^-^ cells from the same patient paired Wilcoxon rank test was used. Survival curves were analyzed by the Kaplan–Meier method. Log-rank test was performed for estimating survival differences between the groups. Cutoff value for separation of individuals into DNAM-1^high^ and DNAM-1^low^ was determined using receiver operating characteristic (ROC) analysis in JMP® Pro as previously described ^13^. Value of highest Youden’s index was used as cutoff. P values of < 0.05 were considered statistically significant. Patients with missing data were excluded from statistical analysis.

## Results

### Patient characteristics

The clinical characteristics of the patient cohort are summarized in Table 1. Our cohort had an almost equal distribution of male and female individuals, with a median age of 63, ranging from 21 to 86 years. Sixteen patients presented with undifferentiated leukemia (according to FAB classification: M0, M1), 18 with immature granulocytic leukemia (M2, M3), 18 with monocytic leukemia (M4, M5) and 10 with erythroleukemia (M6). The majority of patients were diagnosed with primary (53 patients) AML, whereas 9 presented with secondary AML. Risk score allocation according to the National Comprehensive Cancer Network (NCCN) resulted in 22 patients with a favorable risk, 21 patients with an intermediate risk and 9 with poor risk score; for three patients data were not available.

**Table 1:** Clinical characteristics of the patient cohort.

UPN^a^	FAB^b^	DNAM-1 SFI	Age (years)	Sex	WHO^c^	NCCN^d^	WBC^e^ (giga/l)	Hb^f^ (g/dl)	Plt^g^ (giga/l)
UPN1	M5	2.2	58	F	1	0	27.6	8.3	6
UPN2	M6	2.1	70	M	4	1	190.9	7.1	65
UPN3	M1	1.4	40	M	1	2	81.3	10.8	51
UPN4	M2	1.7	86	F	4	3	256.5	7.7	135
UPN5	M0	2.9	85	M	2	2	25.2	9.8	20
UPN6	M4	20.4	64	F	3	0	61.5	7.2	100
UPN7	M4	2.4	71	M	4	1	87.1	7.5	23
UPN8	M3	2.5	46	M	1	0	8.42	9.9	40
UPN9	M6	3.9	69	F	1	3	274.9	7.1	47
UPN10	M1	1.9	67	F	1	0	186.5	6.6	6
UPN11	M0	53.6	81	F	4	1	32.8	7.5	21
UPN12	M1	1.7	27	M	1	0	135.1	8.1	20
UPN13	M3	5.3	58	M	1	0	4.6	7	11
UPN14	M4	1.6	41	F	1	2	112.7	8.5	30
UPN15	M4	2.7	21	M	1	0	125	10.9	24
UPN16	M2	1.2	63	M	1	0	183.3	8.1	31
UPN17	M3	2.2	29	M	1	0	21.6	7.1	61
UPN18	M2	1.2	58	F	2	2	62	7.4	25
UPN19	M6	5.0	63	M	1	1	92.3	10.5	433
UPN20	M5	34.7	35	F	4	1	45.4	8.9	81
UPN21	M6	2.7	78	M	1	2	11.7	8	13
UPN22	M6	1.7	58	M	3	1	87.9	9.5	148
UPN23	M4	2.8	45	F	2	1	448.3	6.6	36
UPN24	M6	3.7	74	M	4	2	239.2	5.7	122
UPN25	M6	14.9	76	M	4	2	169.3	9.9	26
UPN26	M0	1.0	46	M	1	0	60.6	7	39
UPN27	M2	1.7	68	F	4	1	165.7	3.8	222
UPN28	M1	2.5	75	F	1	0	149.7	10.8	174
UPN29	M3	7.1	69	M	1	0	12.7	9.1	73
UPN30	M1	2.3	71	F	4	1	25.8	7.8	50
UPN31	M1	1.0	64	F	1	3	222	9.2	44
UPN32	M1	1.3	78	M	2	2	112.1	9.2	229
UPN33	M5	12.2	37	F	1	1	126.8	9.7	41
UPN34	M6	13.0	81	M	1	0	61.3	11.7	72
UPN35	M2	1.0	47	F	4	3	56.3	9.4	60
UPN36	M1	3.9	62	M	1	1	22.2	12.2	8
UPN37	M5	1.2	23	M	4	1	153.5	6.7	44
UPN38	M1	0.9	76	F	1	0	10.7	7.4	145
UPN39	M5	8.7	68	M	1	1	148.7	9.1	134
UPN40	M3	3.0	58	F	1	0	42.11	8.4	17
UPN41	M2	7.2	71	F	2	1	16.4	8.6	18
UPN42	M2	40.6	79	F	4	2	21.5	7	26
UPN43	M3	1.4	46	F	1	0	21.6	4.4	13
UPN44	M4	26.1	67	F	4	3	315.9	8.2	34
UPN45	M4	9.6	45	M	1	0	26.9	10	24
UPN46	M1	1.0	52	M	1	1	187.5	10.4	31
UPN47	M4	1.5	69	F	1	1	46.3	10.4	34
UPN48	M4	2.3	46	F	1	3	129.3	9.9	211
UPN49	M4	9.7	57	F	4	1	18.94	8.7	128
UPN50	M4	1.7	72	F	4	3	44.6	9.6	69
UPN51	M1	1.8	64	F	4	0	37.1	10	10
UPN52	M4	4.9	54	F	1	1	17.2	10.6	167
UPN53	M1	1.2	34	M	1	0	33.3	10.2	11
UPN54	M2	1.4	83	M	4	1	6.8	11.5	45
UPN55	M6	2.5	53	M	1	0	105.6	8.1	35
UPN56	M0	1.7	49	M	4	1	29.3	12.9	39
UPN57	M2	1.6	67	F	4	1	112.7	9.7	82
UPN58	M3	3.6	65	M	1	0	6.9	8.5	17
UPN59	M6	12.9	22	M	1	0	6.8	8.4	92
UPN60	M2	1.6	64	M	NA^g^	NA	NA	NA	NA
UPN61	M4	21.4	76	F	NA	NA	NA	NA	NA
UPN62	M2	6.2	58	M	NA	NA	NA	NA	NA

^**a**^UPN: unique patient number.

^**b**^FAB: French–American–British classification.

^**c**^WHO: WHO Classification 2008. 1—AML with recurrent genetic aberrations; 2—AML with myelodysplasia-related changes; 3—Therapy-related myeloid neoplasms; 4 AML, not otherwise specified.

^**d**^NCCN: National Comprehensive Cancer Network classification 2018. 0—favorable risk, 1—intermediate risk, 2—poor risk, 3—not defined.

^**e**^WBC: white blood cell count.

^**f**^Plt: thrombocytes.

^**g**^NA: no data available.

### DNAM-1 is expressed on malignant myeloid cell lines and leukemic blasts from AML patients

**Figure 1:**
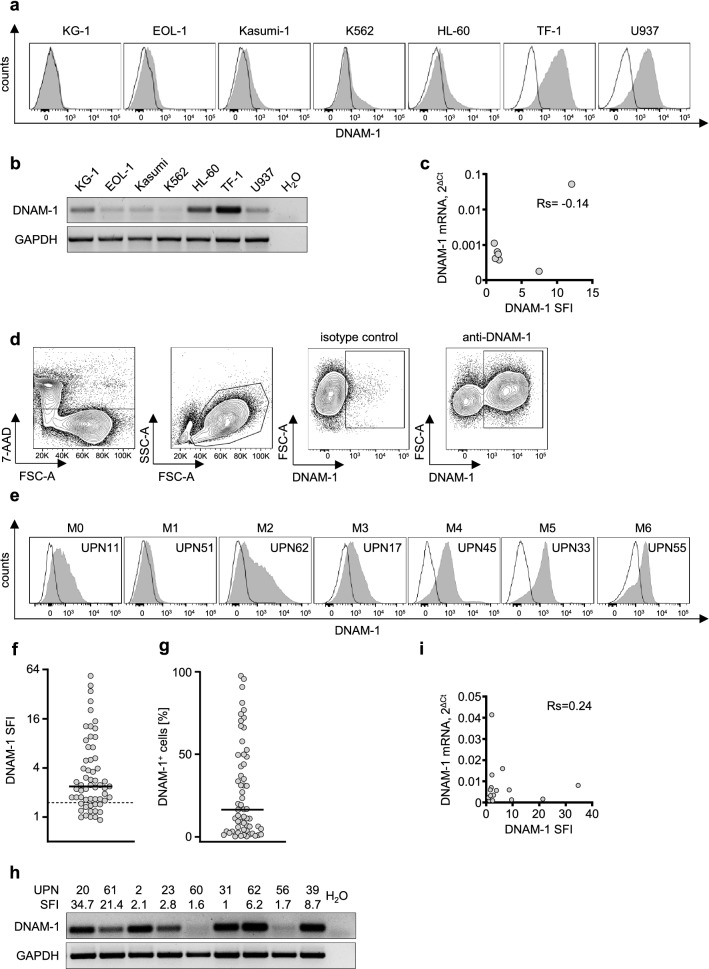
DNAM-1 is expressed on AML cell lines and leukemic blasts from AML patients. (**A**) DNAM-1 surface expression of 7 AML cell lines was analyzed by flow cytometry (shaded peaks, anti-DNAM-1 mAb; open peaks, isotype control). (**B**) Detection of DNAM-1 transcripts in cell lines from (**A**) with RT-PCR is shown. GAPDH was used as a reference gene. (**C**) DNAM-1 mRNA was amplified using quantitative RT-PCR. Abundance of DNAM-1 transcript was calculated using delta-Ct method relative to GAPDH. mRNA expression values (2^∆Ct^) are plotted against specific fluorescence intensity (SFI) values. (D-G) DNAM-1 expression on leukemic blasts from AML patients were analyzed by flow cytometry. (D) Gating strategy of an exemplary AML sample (UPN6) is depicted. Plots with selection for viability (7AAD^−^), mononuclear cells, and DNAM-1 expression are shown. (**E**) Exemplary histograms derived from samples of different FAB-subtypes are shown (shaded peaks, anti-DNAM-1; open peaks, isotype control). (**F**,**G**) DNAM-1 expression on blasts of AML patients (n = 63) was summarized as SFI values (**F**) or proportion of DNAM-1^+^ cells (**G**) (solid line, median; dotted line, SFI = 1.5). (**H**,**I**) DNAM-1 mRNA expression was analyzed by RT-PCR and GAPDH was used as a reference gene. (**H**) Amplification results of exemplary cDNA samples is depicted. Corresponding DNAM-1 SFI values are shown for each sample. (**I**) DNAM-1 mRNA was amplified using quantitative RT-PCR. Abundance of DNAM-1 transcript was calculated using delta-Ct method relative to GAPDH. mRNA expression values (2^∆Ct^) are plotted against SFI values (n = 16). Rs, Spearman correlation coefficient. Full-length gels are presented in Supplementary Fig. 1.

As a first step, we used flow cytometry to determine DNAM-1 surface expression on cancer cell lines representing a wide range of myeloid malignancies: K562 (chronic myeloid leukemia), U937 (pro-myelocytic leukemia), KG-1 (AML M6), EOL-1 (AML M4), Kasumi-1 (AML M2), HL-60 (AML M2), TF-1 (AML M6) (Fig. 1A). We detected no relevant expression in KG-1 and K-562 (SFI > 1.5 as threshold defined for positivity), whereas modest expression was observed with EOL-1, Kasumi and HL-60 cells (SFI = 1.8, 1.7 and 1.7, respectively). TF-1 and U937 cells showed strong DNAM-1 positivity (SFI = 12 and 7.5, respectively). We further confirmed DNAM-1 expression by quantifying mRNA expression in leukemic cells using qRT-PCR (Fig. 1B,C). Notably, DNAM-1 transcript expression showed no correlation with surface levels, which indicates that DNAM-1 surface expression may be regulated posttranscriptionally.

Next, we analyzed DNAM-1 expression on leukemic blasts from AML patients. The gaiting strategy and examples of surface staining are shown in Fig. 1D,E. Again, considering AML specimen with SFI ≥ 1.5 to be DNAM-1 positive, 77% (48 of 62) of all AML cases were found to express substantial DNAM-1 surface levels (Fig. 1F). Individual SFI values of all patients are given in Table 1. The number of positive cells within the leukemic population of individual patients varied considerably, ranging from 0% to almost 100% (Fig. 1G). When DNAM-1 expression in the AML samples was quantified using RT-PCR (Fig. 1H,I), alike with the cell lines, DNAM-1 transcripts were detectable in some samples without relevant surface expression on leukemic cells. In line, no correlation between DNAM-1 mRNA levels quantified by qRT-PCR and surface expression was observed (Fig. 1I). These results further hint at posttranscriptional regulation of surface DNAM-1 levels. Together, our data reveal widespread expression of DNAM-1 in a substantial number of AML cases.

### DNAM-1 stimulates cytokine release by AML cells

**Figure 2:**
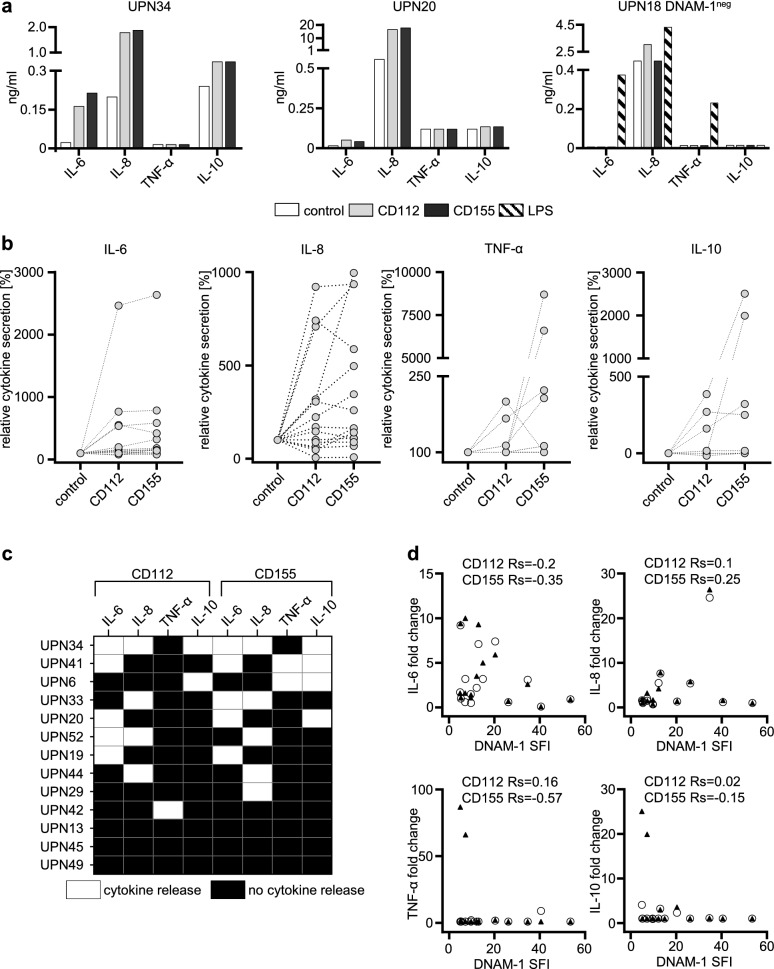
DNAM-1 signaling induces cytokine release from AML cells. Cytokine secretion by AML samples upon incubation with immobilized CD112-, CD155-Fc fusion proteins or hIgG1 (control) was measured by ELISA. (**A**) Representative results of cytokine measurements form 2 exemplary DNAM-1-positive AML samples (UPN34 and UPN20) and one DNAM-1-negative sample (UPN 18, similar results were obtained with 5 DNAM-1-negative samples in total) are depicted. Mean values of duplicate measurements are shown. LPS was used as non-specific stimulator of cytokine production (positive control). (**B**) Cytokine secretion in 13 AML samples was normalized to the hIgG-treated control. Individual AML samples are shown by connecting lines. (**C**) Overview of cytokine secretion by the samples tested is shown. Cytokine production > 3 times higher than hIgG-treated cells is depicted in white. (**D**) Correlation of cytokine secretion with DNAM-1 surface levels (SFI) is shown. Rs, Spearman correlation coefficient. Triangles: CD155, circles: CD112.

As interaction of DNAM-1 with its ligands promotes activation and cytokine secretion in various cell types^14–16^, we next set out to determine whether DNAM-1 was functional in AML cells. To this end we analyzed cytokine production in cultures of primary AML specimens upon stimulation with the two different DNAM-1 ligands CD112 and CD155. We selected thirteen AML samples with high DNAM-1 expression on leukemic cells (SFI ≥ 5, 10–90% positive cells, median positivity 70%) and incubated them on immobilized recombinant human DNAM-1 ligands (rhCD112-Fc or rhCD155-Fc) or isotype control (human IgG1) for 24 h. Afterwards secretion of the immunomodulatory cytokines IL-6, IL-8, TNF-α and IL-10 that are implicated in AML pathophysiology^17,18^ were analyzed by ELISA. Exemplary cytokine profiles obtained with 2 DNAM-1-positive (UPN34 and UPN20) and 1 negative specimen (UPN18) as control are shown in Fig. 2A. Treatment with DNAM-1 ligands potently stimulated cytokine release in the positive samples, whereas no effect was observed with DNAM-1-negative leukemia. Stimulation with LPS, a non-specific inducer of cytokine secretion in leukemia cells^19^, induced release of cytokines in DNAM-1 negative samples, confirming that effects observed with positive leukemic cells were specifically mediated by DNAM-1 signaling (Fig. 2A). Substantial interindividual differences with regard to cytokine release were observed (Fig. 2B,C). Out of 10 samples that responded to stimulation, none responded with release of all four cytokines. In one sample, production of IL-6, IL-8 and IL-10 was observed, whereas 5 and 4 samples secreted at least 2 and only one of the cytokines, respectively (Fig. 2C). Of note, no correlation of DNAM-1 expression with release of the cytokines was observed (Fig. 2D).

Together, these findings demonstrate that DNAM-1 is functional in AML cells with substantial interindividual differences with regard to the consequences of signaling that may differentially contribute to the cytokine milieu.

### Correlation of DNAM-1 expression with clinical characteristics of AML patients

**Figure 3:**
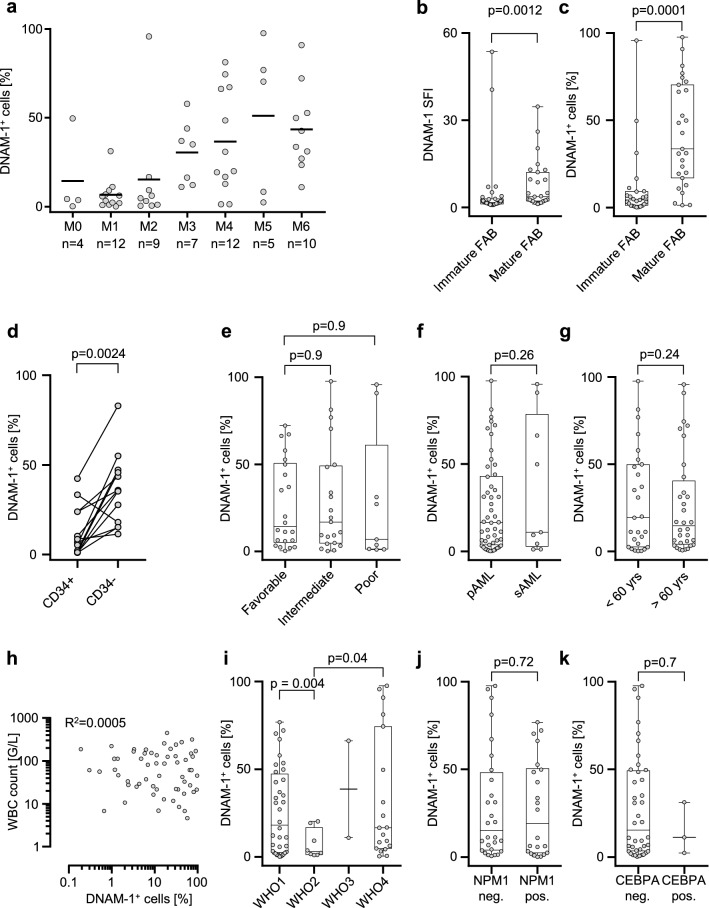
DNAM-1 expression is associated with clinical parameters. (**A**) Proportion of DNAM-1^+^ cells in AML samples grouped according to the FAB subtype; median and individual values are shown. (**B**–**C**) Distribution of DNAM-1 expression in immature (M0-2) versus mature (M4–M6) FAB subtypes is shown; statistical significance was assessed using Mann–Whitney test. (D) Distribution of DNAM-1^+^ cells among CD34^+^ and CD34^-^ populations of AML blasts is depicted. Values derived from the same sample are connected with lines; n = 12, statistical significance was assessed using Wilcoxon paired rank test. (**E**–**G**) Distribution of DNAM-1 expression (% of positive cells) across NCCN risk groups (**E**), primary (pAML) and secondary (sAML) cases (**F**), and two age groups (**G**) is demonstrated. (**H**) Correlation of DNAM-1 expression with WBC counts is illustrated. (**I**–**K**) Distribution of DNAM-1 expression across AML types based on WHO classification (**I**), AML cases positive or negative for NPM1 and CEBPA mutations (**J**,**K**) is shown. Statistical significance was assessed using Mann–Whitney (**D**,**G**,**J**,**K**), Kruskal–Wallis (**E**,**F**) or Brown–Forsythe and Welch ANOVA (**I**) tests. Rs, Spearman correlation coefficient.

Next, we analyzed association of DNAM-1 expression with clinical characteristics of AML patients (n = 59, 3 patients were not analyzed due to lack of data on WHO classification, NCCN risk score and blood counts). First, we correlated the intensity of DNAM-1 surface staining with morphological characteristics according to the FAB classification. The fractions of positive (SFI ≥ 1.5) samples among analyzed patients with different FAB types were as follows: M0, 3 of 4 (75%); M1, 6 of 12 (50%); M2, 5 of 9 (56%); M3, 6 of 7 (86%); M4, 11 of 12 (92%); M5, 4 of 5 (80%) and M6, 10 of 10 (100%). The smallest mean DNAM-1 positivity (SFI > 1.5) was found among M1 subtype samples (6.7%), the highest positivity was observed with FAB M5 samples (mean positive cell fraction 51%) (Fig. 3A). Expression of DNAM-1 was significantly associated with leukemia cell maturity: specimens from differentiated FAB types M4-M6 demonstrated stronger positivity and higher SFI values than M0-M3 subclasses (p < 0.0001 and p = 0.0012, respectively) (Fig. 3B,C).

To further investigate the correlation of DNAM-1 expression with maturation characteristics, we selected cases with abundant CD34-positive (> 10% CD34^+^ cells) and assayed DNAM-1 expression on CD34^+^ versus CD34^-^ cells. We found that DNAM-1 positive cells were enriched in the CD34^-^ population (Fig. 3D, p = 0.0024), further confirming that DNAM-1 expression is associated with a differentiated phenotype.

Analysis of the correlation of DNAM-1 expression with factors affecting prognosis did not reveal an association for NCCN risk class (Fig. 3E), primary versus secondary disease (Fig. 3F) and age (< 60 vs. ≥ 60 years) (Fig. 3G). Moreover, no relationship between DNAM-1 positivity and white blood count was observed (Fig. 3H). Analysis of correlation of DNAM-1 expression with AML subtypes according to WHO criteria revealed significantly lower DNAM-1 expression among AML cases classified as “AML with myelodysplasia-related changes” compared to “AML with recurrent genetic aberrations” and “AML, not otherwise specified” (p = 0.004 and p = 0.04 respectively) (Fig. 3I). Expression of DNAM-1 did not show any correlation with NPM1 and CEBPA mutations (Fig. 3J,K).

### DNAM-1 expression correlates with survival of AML patients

**Figure 4:**
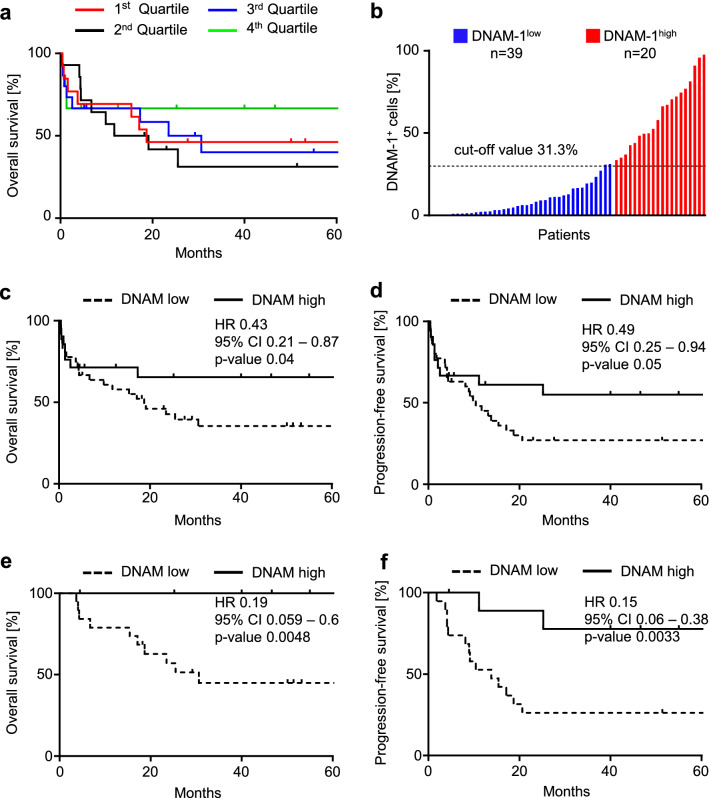
Prognostic evaluation of DNAM-1 in AML. (**A**) Overall survival of patients divided into quartiles according to DNAM-1 expression is depicted. (**B**) Separation of the cohort into DNAM-1^high^ and DNAM-1^low^ groups using cut-off value of 31.5% positive cells is illustrated. (**C**,**D**) Overall and progression-free (**E**,**F**) survival of patients assigned to the DNAM^high^ and DNAM^low^ groups based on the cut-off value 31.3%; (**C**,**D**)—all patients, (**E**,**F**)—patients subjected to allogeneic stem cell transplantation (HSCT) is shown; statistical significance was assessed using log-rank test.

To determine whether DNAM-1 expression has prognostic value in AML, we correlated DNAM-1 surface positivity with survival of 59 patients; for 3 individuals survival data were not available. For initial analysis, individuals were divided into quartiles according to DNAM-1 expression, and overall survival (OS) in each quartile was assessed. Kaplan–Meier analysis did not reveal any difference in survival between the four groups (p = 0.49) (Fig. 4A). Next, we employed receiver operating characteristic (ROC) analysis to determine a predictive cut-off for separation of patients into groups. A cut-off value of 31.3% was selected to subdivide AML samples into DNAM-1^high^ (n = 20) and DNAM-1^low^ (n = 39) groups (Fig. 4B). Upon separation of patients according to this threshold, DNAM-1^high^ patients displayed significantly longer OS (hazard ratio 0.43, p = 0.04, Fig. 4C). Progression free survival (PFS) analysis did not reveal statistically significant differences between the groups (p = 0.05), but demonstrated a similar trend (Fig. 4D). When only patients who received hematopoietic stem cell transplantation (HSCT) were included in the analysis, an even more pronounced difference between the groups was revealed: individuals in the DNAM-1^high^ group showed significantly longer OS and PFS (p = 0.0048 and 0.0033 respectively) (Fig. 4E,F). These results identify DNAM-1 as potential prognostic marker in AML.

## Discussion

DNAM-1 is an important mediator of effector functions in NK cells and T cells. Besides cytotoxic lymphocytes, functionally relevant DNAM-1 expression was described on B cells, but also on healthy cells of the myeloid lineage like monocytes and platelets^7,8,20,21^. Whereas DNAM-1 is mostly known for its cytotoxicity-stimulating properties in NK cells and T cells, in myeloid cells it was reported to influence migration^8^. Beyond healthy cells, DNAM-1 expression was also reported for chronic lymphocytic leukemia (CLL)^22^. Whether DNAM-1 is expressed in malignant myeloid cells and whether it contributes to disease pathophysiology is so far unknown.

Here we report that DNAM-1 is expressed in cell lines of myeloid lineage and primary leukemic cells of AML patients. When we investigated the functionality of the DNAM-1 in AML cells, we found that interaction with its ligands resulted in production of the cytokines IL-6, IL-8, TNF-α and IL-10. This is in line with reports that interaction of DNAM-1 on cytotoxic cells with CD155 or CD112 triggers signaling via immunoreceptor tail tyrosine (ITT)-like motifs and stimulates cytokine secretion^14,23^. No correlation between the extent of cytokine release and the surface levels of DNAM-1 was observed, suggesting that the signaling capacity of the receptor is influenced by other factors, for example by co-expression of inhibitory molecules such as CD96^24,25^.

IL-6 and IL-8 are implicated to affect cellular survival in various cancer entities including AML^26–31^ and associate with disease outcome^32–34^. IL-10 reportedly influences proliferation and cytokine production by AML cells ^26,35,36^. Elevated plasma levels of IL-10 correlate with better survival of patients. For TNF-α controversial effects in AML pathogenesis have been reported^37^. Notably, variable patterns of cytokine release upon DNAM-1 signaling were observed. Whereas IL-6 or IL-8 were released by more than a half of the samples tested, secretion of IL-10 and TNF-α was observed less frequently. Likely the ultimate effect of DNAM-1 signaling may be is determined by combinatorial action of several secreted factors. Regarding the association of DNAM-1 expression and clinical parameters, we found that more mature AML cells displayed higher DNAM-1 positivity compared to immature FAB subclasses. Thus, DNAM-1 may serve as additional maturation marker upon immunophenotyping. Further in line with the association of DNAM-1 positivity and a more differentiated phenotype, we observed substantially higher expression on mature CD34-negative subpopulations of leukemic cells.

Various activating immune receptors (e.g. Fc-receptors) are expressed on AML cells^19,38–40^, and expression associates with prognosis^41,42^. To evaluate whether DNAM-1 expression has prognostic relevance in AML, we performed multivariate analysis with several factors influencing disease outcome. We did not identify an association between DNAM-1 expression and age, primary/secondary AML, risk profile according to the NCCN guideline and NPM1 and CEBPA mutations. Likewise, no significant differences in DNAM-1 expression between FAB subtypes with unfavorable (M0, M6) and favorable (M1-M5) prognosis were detected^43–46^. However, decreased DNAM-1 expression was associated with cases with myelodysplasia related changes when compared to other subtypes.

When we employed ROC analysis and separated AML patients in two groups according to a defined cut-off value of 31.3% of DNAM-1 positive cells, a significant correlation of high DNAM-1 surface levels with better OS was observed. Notably, high DNAM-1 expression was also associated with improved OS and PFS in patients after HSCT.

The role of NK cell-expressed DNAM-1 in recognition and killing of AML cells is well established. Interaction of DNAM-1 with its ligands CD112 and CD155 on AML cells is required for NK cell activation and cytotoxicity^47^. Attenuated expression of DNAM-1 on NK cells or DNAM-1 ligands on AML cells was suggested as an immune escape mechanism^48^. In agreement with this, higher expression of DNAM-1 on NK cells was associated with increased survival in AML patients^49^. The data presented here show that elevated DNAM-1 expression on AML cells themselves was also associated with improved survival of AML patients. So far, it remains unclear to which extent improved survival of individuals in the DNAM-1^high^ group can in fact be attributed to engagement of the signaling axis or is rather an epiphenomenon of the association with more differentiated status of AML cells. This is even more since, as stated above, DNAM-1 expression was found to be associated with a CD34-negative blast phenotype and survival of patients negatively correlates with the amount of CD34-positive leukemic cells^50^.

To conclude, we here provide first evidence that DNAM-1 is functionally expressed on AML cells and expression correlates with survival of the patients. Based on the data reported, confirmatory studies in larger cohorts are warranted and hold promise to establish DNAM-1 expression as a prognostic marker for risk stratification and ultimately to improve outcome for AML patients.

## Supplementary Information

Supplementary Information 1.

## Data Availability

The datasets generated during and/or analysed during the current study are available from the corresponding author on reasonable request.

## Abbreviations

DNAM-1: DNAX accessory molecule-1
FAB: French–American–British classification
Hb: Hemoglobin
ITT-motif: Immunoreceptor tail tyrosine motif
NA: No data available
NCCN: National Comprehensive Cancer Network classification 2018
OS: Overall survival
PFS: Progression-free survival
Plt: Thrombocytes
qRT-PCR: Quantitative reverse transcription PCR
ROC: Receiver operating characteristic
SFI: Specific fluorescence index
UPN: Unique patient number
WBC: White blood cell count
WHO: WHO Classification 2008
