# Supplementary figures and images for: DNAM-1/CD226 is functionally expressed on acute myeloid leukemia (AML) cells and is associated with favorable prognosis
Source: Sci Rep. 2021 Sep 9;11:18012. doi: 10.1038/s41598-021-97400-6 (PMC8429762; doi:10.1038/s41598-021-97400-6)

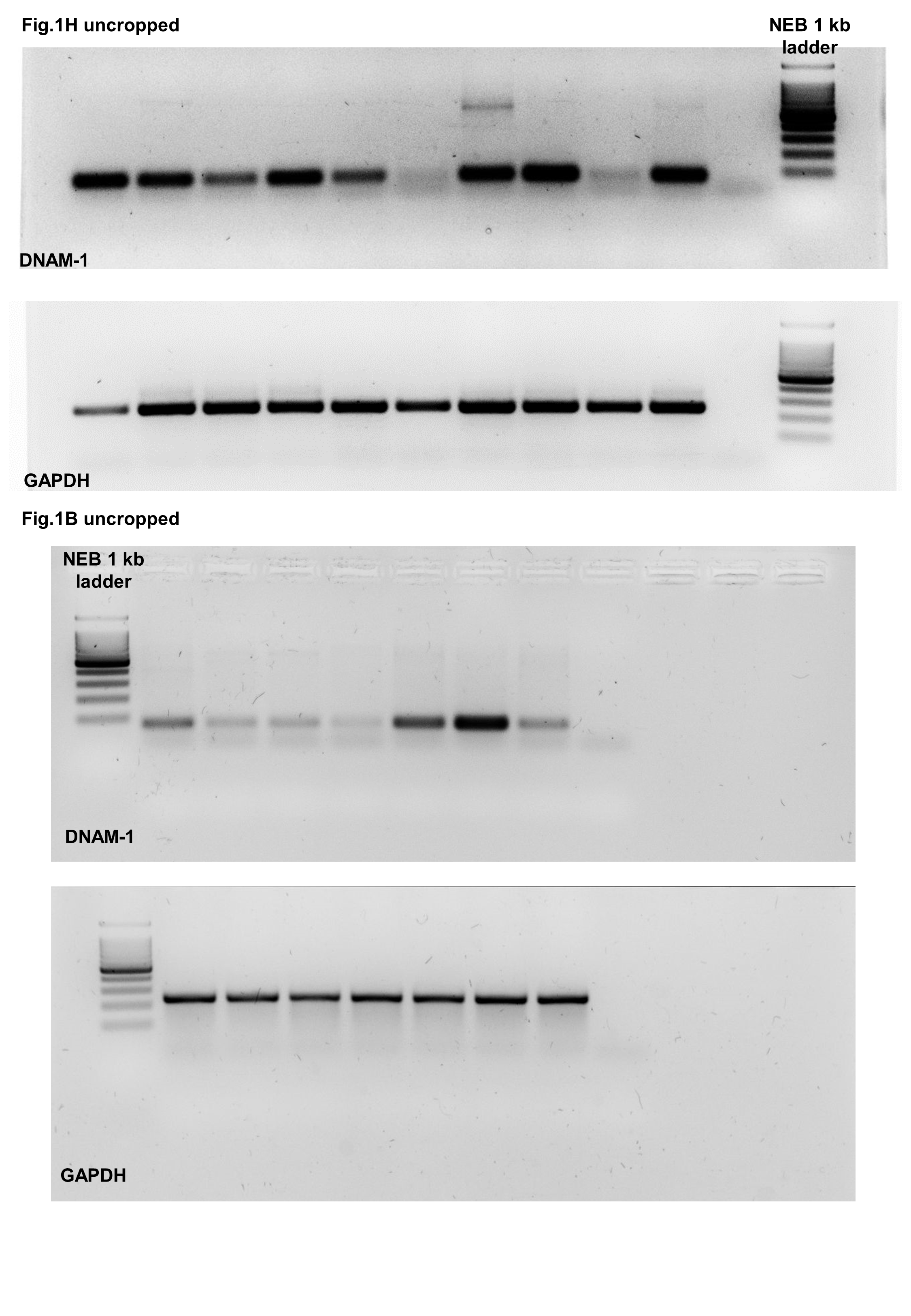

Supplement: Supplementary file 1 — Supplementary Information 1. [file 41598_2021_97400_MOESM1_ESM.tif]
